# How does intraarticular dexmedetomidine injection effect articular cartilage and synovium? An animal study

**DOI:** 10.1186/s12871-020-01148-x

**Published:** 2020-09-17

**Authors:** Başak Akça, Aysun Ankay Yılbaş, Filiz Üzümcügil, Berkem Büyükakkuş, Elham Bahador Zırh, Dilara Zeybek, Fatma Sarıcaoğlu

**Affiliations:** 1 Department of Anesthesiology and Reanimation, Hacettepe University School of Medicine, Sıhhiye, 06230 Ankara, Turkey; 2 Department of Histology and Embryology, Hacettepe University School of Medicine, Ankara, Turkey

**Keywords:** Dexmedetomidine, Injection, Intraarticular, Rats, Local anesthetics, Joint, Knee

## Abstract

**Background:**

Intraarticular injections are widely used to provide pain relief after arthroscopic procedures and minimize the use of opioids. Dexmedetomidine has been proven to potentiate pain relief and postpone the demand for the first analgesic drug when it is used intraarticularly following arthroscopic knee procedures. However, the effects of dexmedetomidine on articular structures have not yet been evaluated. Our aim was to determine the effects of intraarticular dexmedetomidine injection on articular structures such as cartilage and synovium.

**Design:**

Animal study.

**Methods:**

Twenty adult rats (Sprague-Dawley) were enrolled in the study. Following appropriate aseptic and anesthetic conditions, dexmedetomidine (100 mcg/ml) (0.25 ml) was injected into the right knee joint (the study group) and normal saline solution (0.25 ml) into the left knee joint (the control group) of the rats. Four rats were sacrificed from each group on days 1, 2, 7, 14, and 21, and knee joint samples were obtained. Histologists evaluated the articular and periarticular regions and the synovium using histological sections, and a five-point scale was used to grade the inflammatory changes in a blinded manner.

**Results:**

The groups were found to be similar in terms of median congestion scores, edema and inflammation scores, subintimal fibrosis, neutrophil activation and cartilage structure at each of the time intervals.

**Conclusion:**

In our placebo-controlled, in vivo trial, the intraarticular use of dexmedetomidine seemed to be safe with respect to the studied histopathological parameters. However, complementary studies investigating the histopathological effects, analgesic dosage and adverse effects of dexmedetomidine on damaged articular structure models are needed.

## Background

Knee arthroscopy is a commonly performed procedure for both the diagnosis and treatment of internal structures of the knee. Arthroscopic procedures, especially knee arthroscopy, are associated with serious postoperative pain caused by irritation of the free nerve endings of the synovial tissue, anterior fat pad, and joint capsule during surgical excision and resection [1–3]. Adequate analgesia is the key to patient satisfaction and low morbidities [4]. Multimodal analgesia techniques (systemic medications and peripheral or central nerve blockades) have been used, but none of these techniques are free of complications or limitations.

The intraarticular route targets peripheral receptors in the joint and delivers analgesia locally with minimum side effects after knee joint surgery [5].

Intraarticular injections of local anesthetics or several other adjuvants have been proven to alleviate postoperative pain; however, the histopathological effects of these drugs on chondrocytes and synovium have been neglected most of the time, with a lack of research into their possible effects on articular tissues. Many human trials investigating the analgesic effects of adjuvant drugs were conducted prior to animal studies and evaluated the effects of the adjuvant drug on the native tissues; however, subsequently, certain adverse effects of the local anesthetics (bupivacaine, lidocaine) were reported in in vitro/in vivo trials [6–8].

Dexmedetomidine is a highly selective α2-adrenoceptor agonist (eight times more selective than clonidine) and is a commonly used sedative-hypnotic, anxiolytic, and analgesic agent. There has been great interest in the intraarticular use of dexmedetomidine in humans [3, 9, 10], but the effects of dexmedetomidine on articular tissues remain controversial. Some trials have shown protective effects against apoptosis induced by anesthetic drugs [11, 12], while conversely, high doses of dexmedetomidine have been found to contribute to apoptosis in human neutrophils [13]. However, dexmedetomidine is not licensed by our country or by the United States Food and Drug

Administration (FDA) for intraarticular usage, and the manufacturers cannot provide any data regarding whether this route of administration is toxic to internal structures of the knee.

In this study, our aim was to determine the histopathological effects of intraarticular dexmedetomidine injection on internal articular structures such as cartilage and synovium.

## Methods

### Patient and public involvement

No patients were involved.

After obtaining approval from the Animal Experimentations Local Ethics Board of Hacettepe University (approval number: 52338575–47), this study was conducted in accordance with certain binding guidelines (European Union Strategy for the Protection and Welfare of Animals).

We enrolled twenty male Sprague-Dawley rats with a mean age of 12 months and weighing 300–350 g. Rats were obtained commercially from a private source (Kobay A.Ş, Ankara/TURKEY-www.kobay.com.tr). Rats were housed in appropriate pathogen-free conditions at a temperature of 20 °C–24 °C (in separate cages, enough for normal activity). The light cycle was fixed at 12 h (12 h light and 12 h dark). All rats received standard food and water ad libitum.

All rats were anesthetized with an intraperitoneal ketamine (75 mg/kg) injection. Under aseptic conditions, 0.25 ml dexmedetomidine (100 mcg/ml) (group D) was injected into the right knee joint, and 0.25 ml normal saline (the control group, group C) was injected into the left knee joint of the rats. In our previous study, we tested the injection technique and determined the intraarticular injection and the volume of the injection by injecting the same amount (0.25 ml) of methylene blue into both knees of an anesthetized rat [14]. After the injections, postoperative care was provided, and then all the rats were placed in their cages. Four rats from each group were sacrificed under ketamine anesthesia (120 mg/kg intraperitoneally) by cervical dislocation as described in “AVMA Guidelines for the Euthanasia at Animals [15]” on days 1, 2, 7, 14, and 21, and knee joint samples were obtained.

The samples were fixed with 10% buffered formalin for 2 weeks and decalcified in “De Castro” solution for 6 weeks at room temperature as in a previous trial [14]. Routine light microscopy was used to assess the decalcified samples. The decalcified samples were processed for routine light microscopy. Briefly, the samples were dehydrated and embedded in paraffin. Five-micrometer-thick serial sections were cut and stained with hematoxylin–eosin and Masson’s trichrome stains according to standard procedures. The sections were examined under a light microscope (Leica DM 6000B) and photographed with a DFC490 digital camera (Leica, Wentzler, Germany).

The histological evaluation of all the articular/periarticular regions and the synovium was performed by two separate histologists in a blinded manner. A four-point scale was used to grade the inflammatory changes in four fields per section and four sections per knee. The histological sections of the periarticular regions and synovium were examined for edema, neutrophil infiltration and congestion. Inflammatory changes were evaluated according to a four-point scale (0/1/2/3 = normal/mild/moderate/severe). Synovial hyperplasia was scored according to the ranking of synovitis (0: single layer of synovium, 1: two layers of synovium, 2: three layers of synovium, 3: four layers of synovium). The percentage of fibrotic tissue was evaluated in Masson’s trichrome-stained sections of loose joint tissue. Fibrotic tissue < 10% was scored as 0, 10–30% fibrotic tissue as 1, 30–50% fibrotic tissue as 2, and > 50% fibrotic tissue as 3.

Changes in the structure of the joint cartilage were evaluated using a modified Mankin score [16] with four parameters as in a previous trial [16] (Table 1) and were graded according to a five-point scale in a blinded manner [14].

**Table 1:** Modified Mankin Scoring System

**Cartilage structure**	
a. Normal	0
b. Surface irregularities	1
c. Pannus + irregularities	2
d. Clefts to the transitional zone	3
e. Clefts to the radial zone	4
f. Clefts to the calcified zone	5
g. Complete disorganization	6
**Cartilage cells**	
a. Normal	0
b. Diffuse hypercellularity	1
c. Cloning	2
d. Hypocellularity	3
**Staining with Masson’s Trichrome**	
a. Normal	0
b. Slight reduction	1
c. Moderate reduction	2
d. Severe reduction	3
e. No staining	4
**Tidemark integrity**	
a. Intact	0
b. Destroyed	1

### Statistical analysis

IBM SPSS Statistics version 17.0 software (IBM Corporation, Armonk, NY, USA) was used for data analysis. Ordinal data are expressed as the median (min–max). The Wilcoxon signed rank test was used to evaluate the statistical significance of the differences in histopathological scores between the dexmedetomidine and saline groups. To control Type I errors due to multiple comparisons, the Bonferroni correction was applied. Therefore, the statistical significance level was set at *p* < 0.01.

### Sample size estimation

Four knee samples in each group were required to show a difference of 65% in inflammation scores in the first 2 days (power 80%, α = 0.05), as determined from our previous studies [16–18]. To sacrifice the minimal available number of rats, we decided to use the right knee of each rat as the study group and the left knee as the control group. Using this technique, we also maintained overall homogeneity among the rat groups. As a result, we ended with a total sample size of twenty rats.

## Results

Both joints of twenty rats (a total of forty) were examined. The results of the histopathologic evaluation of each joint are shown in Table 2.

**Table 2:** Histopathological scores regarding days

	**Group D**	**Group C**	***p value †***
**Congestion**			
*1st day*	1 (1–2)	0.5 (0–1)	0.180
*2nd day*	1 (0–1)	1 (1–1)	0.317
*7th day*	1 (0–1)	0 (0–1)	0.083
*14th day*	1 (0–1)	0 (0–1)	0.157
*21st day*	0 (0–1)	0 (0–0)	0.317
**Edema**			
*1st day*	1 (1–1)	1 (0–1)	0.317
*2nd day*	1 (0–1)	1 (1–1)	0.317
*7th day*	0 (0–0)	0 (0–1)	0.317
*14th day*	0 (0–0)	0 (0–0)	–
*21st day*	0 (0–1)	0 (0–0)	0.317
**Neutrophil infiltration**			
*1st day*	2.5 (2–3)	1 (0–1)	0.059
*2nd day*	2 (1–3)	2 (1–2)	0.564
*7th day*	0.5 (0–1)	0 (0–1)	0.317
*14th day*	0 (0–1)	0 (0–0)	0.317
*21st day*	0 (0–1)	0 (0–0)	0.317
**Cartilage structure**			
*1st day*	0 (0–1)	0 (0–0)	0.317
*2nd day*	0 (0–1)	0 (0–0)	0.317
*7th day*	0 (0–1)	0 (0–0)	0.317
*14th day*	0 (0–1)	0 (0–0)	0.317
*21st day*	0 (0–1)	0 (0–0)	0.317

Data are shown as the median (min–max), † Wilcoxon sign rank test; according to the Bonferroni correction, *p* < 0.010 was considered statistically significant.

Macroscopic hematoma was not observed in any of the knees.

Mild congestion was evident in the saline group (Group C) at days 1 and 2, while in the dexmedetomidine group (Group D), mild congestion was observed until day 14. There were no significant differences between groups, compared to the saline group, in terms of congestion.

Mild edema was observed at days 1 and 2 in both groups.

**Fig. 1:**
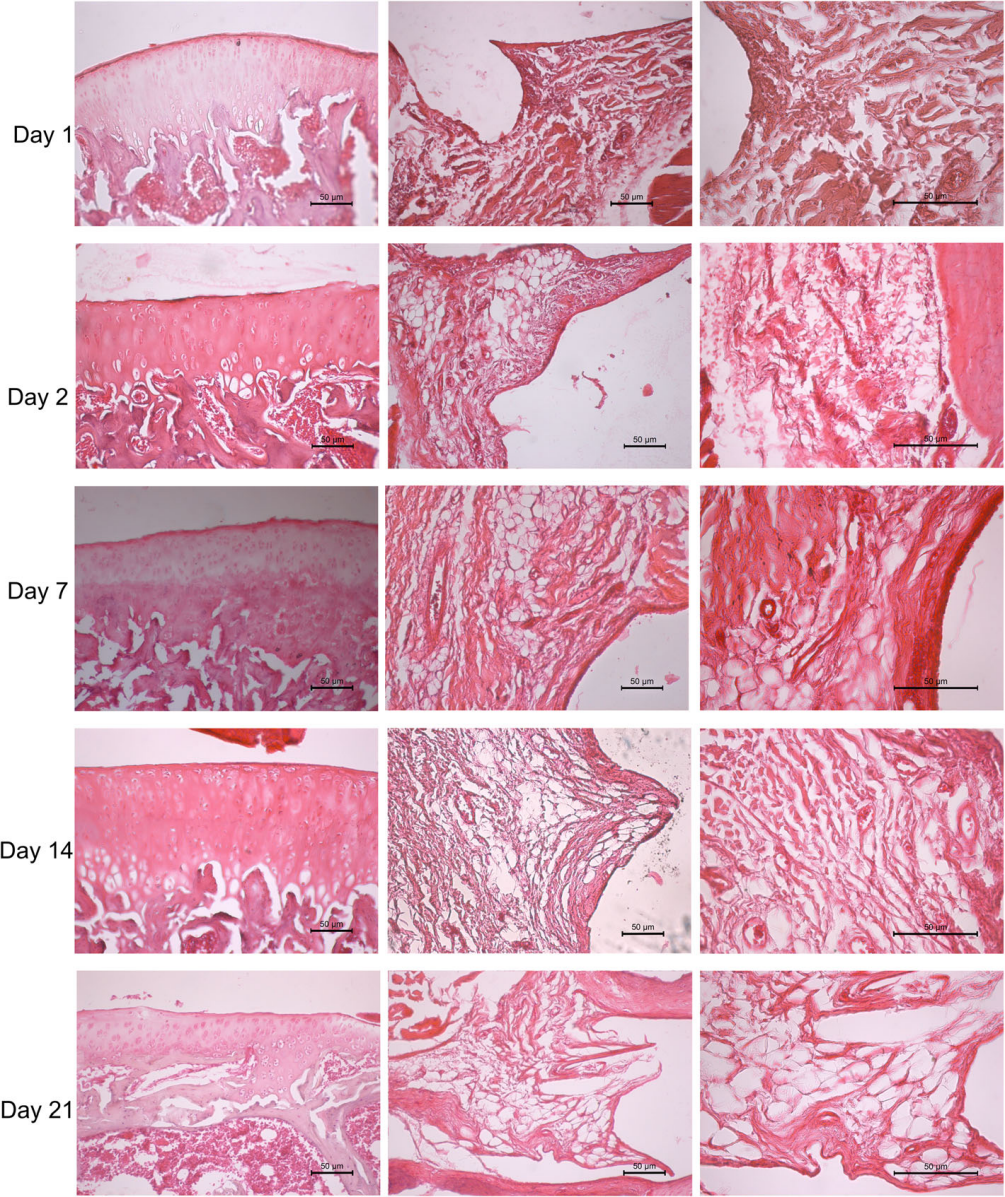
The knee joints and synovium of saline administrated group. No erosion of cartilage surface at all time intervals. Mild congestion and edema at day of 1 and 2. Moderate neutrophil infiltration in saline group at day of 2. Hematoksilen-eosin, Scale bar: 50 μm

**Fig. 2:**
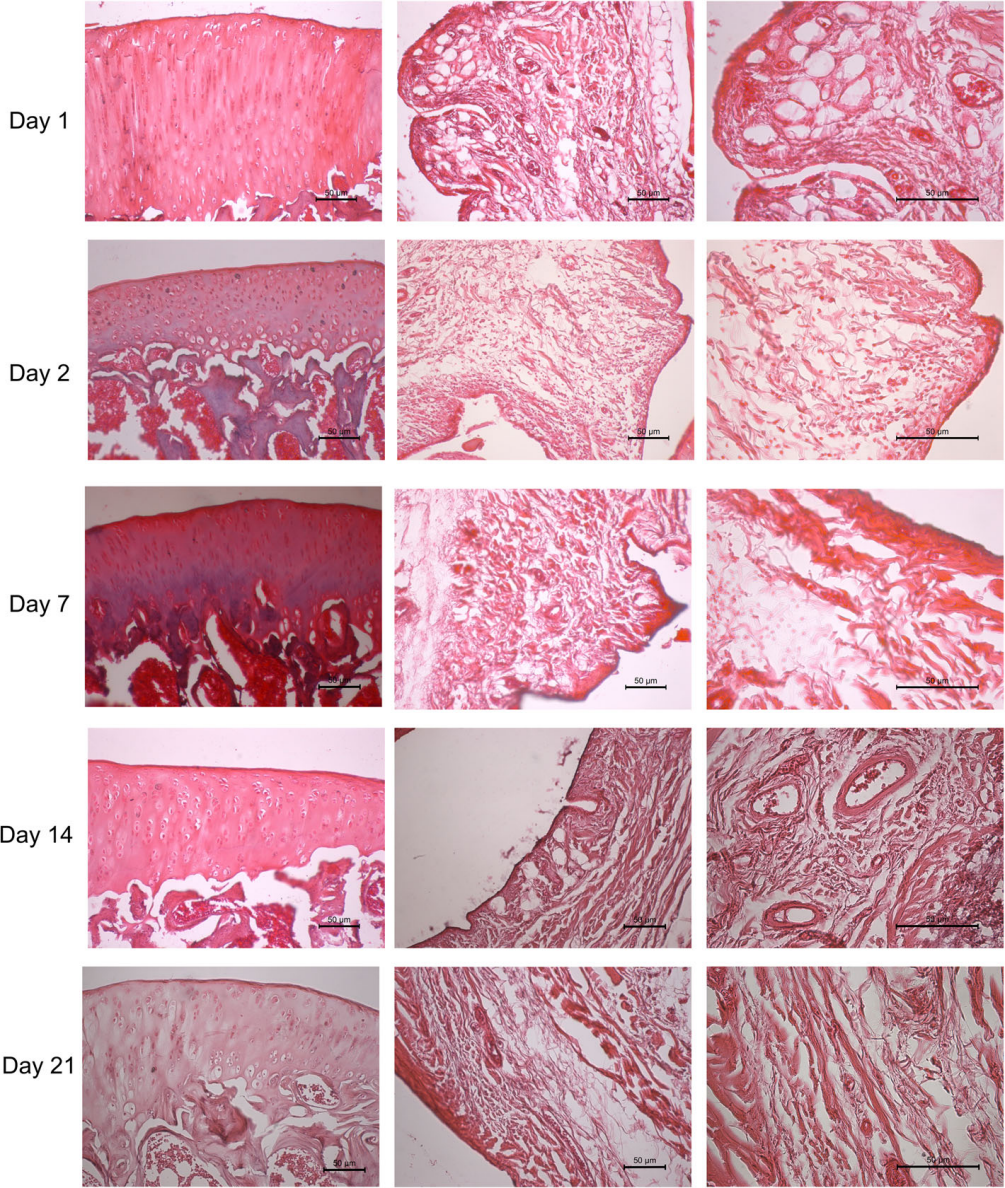
The knee joints and synovium of dexmedetomidine administrated group. Intact cartilage surfaces without erosion at all time intervals. Mild congestion in dexmedetomidine administrated group at day of 1, 2, 7 and 14. Mild edema at day of 1 and 2. Severe neutrophil infiltration at day of 1 and mild neutrophil infiltration at day of 7. No inflammation was observed in synovium at 14 and 21 days in this group. Single row of synoviocytes surrounding the loose connective tissue was seen. Hematoksilen-eosin, Scale bar: 50 μm

On day 1, severe neutrophil infiltration was observed in the dexmedetomidine group. Neutrophil infiltration decreased on subsequent days, and slight neutrophil infiltration was determined at day 7, with no infiltration at days 14 and 21. In the saline group, neutrophil infiltration was mild at day 1 and moderate at day 2. Moreover, at days 7, 14, and 21, there was no neutrophil infiltration in the synovium in the saline group (Fig. 1) (Fig. 2).

**Fig. 3:**
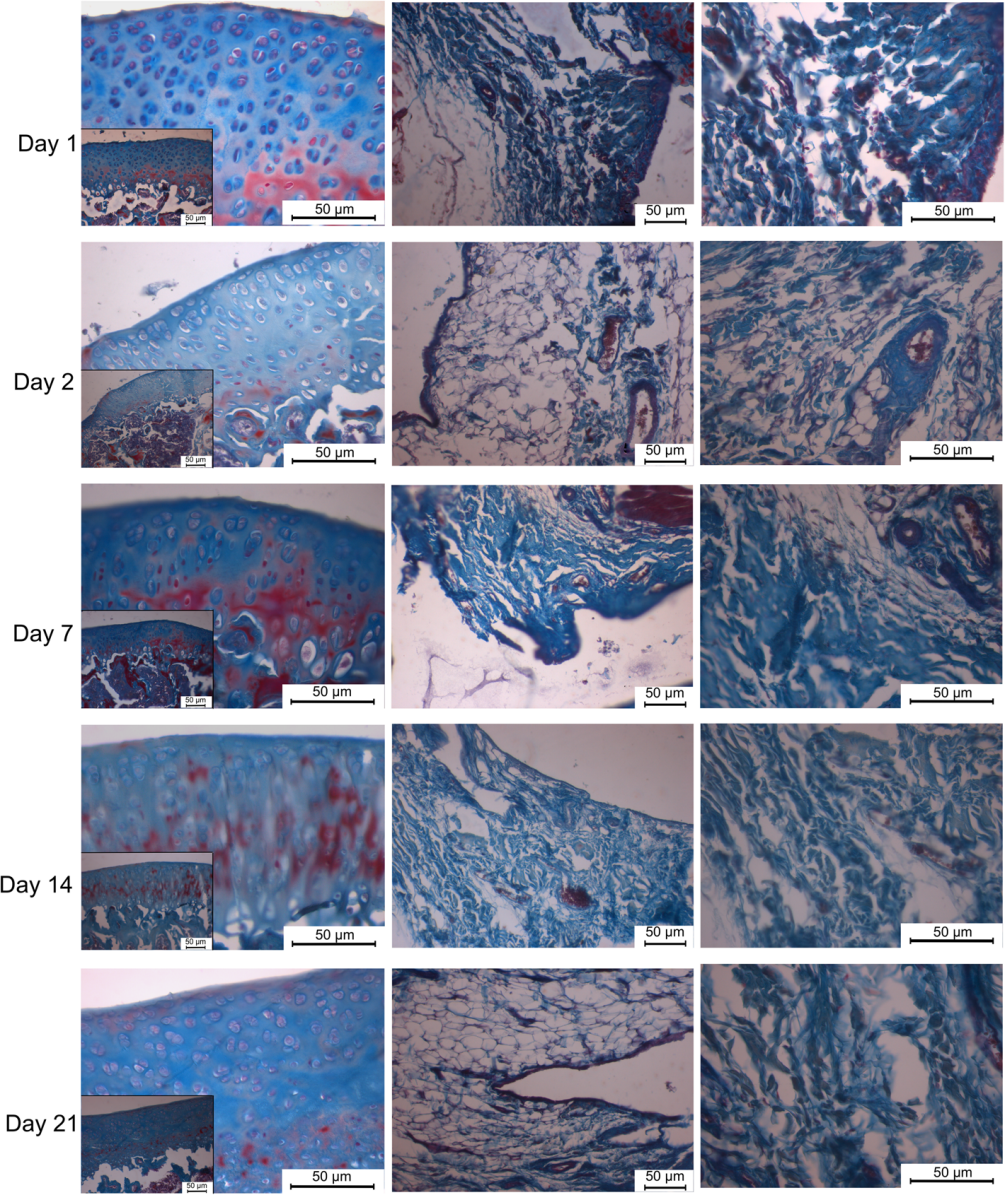
Articular cartilage with normal structure and normal cell morphology in saline group. Synovial membrane composed of single row of synoviocytes lying over loose connective tissue composed of collagen fibers and adipocytes. Masson’s trichrome**.** Scale bar: 50 μm

**Fig. 4:**
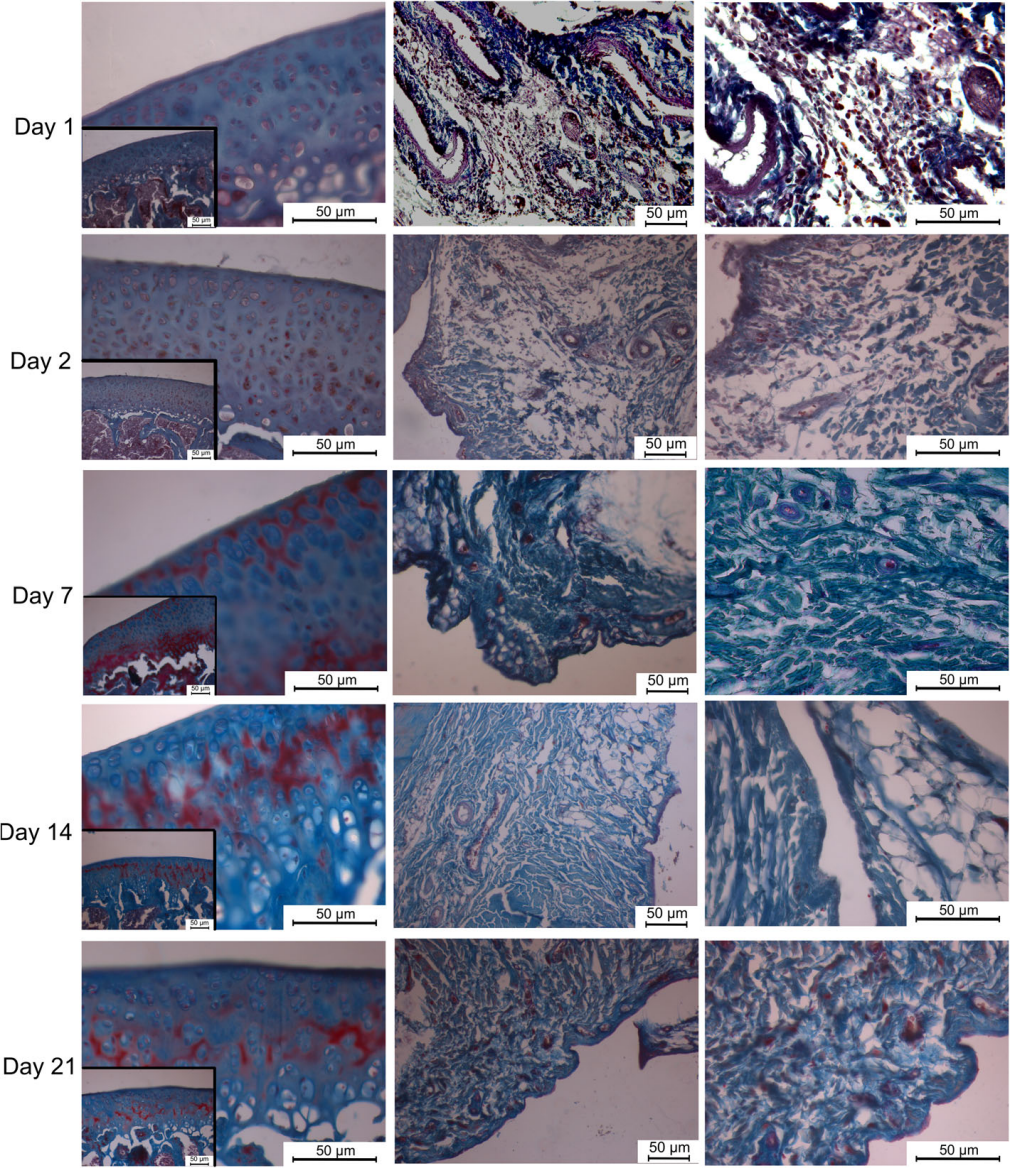
Articular cartilage with normal structure and normal cell morphology in dexmedetomidine group. Moderate neutrophil infiltration in synovium at day of 2. Single row of synoviocytes and collagen fibers in synovium at day of 21. Masson’s trichrome**.** Scale bar: 50 μm

The thickness of the synovial membrane was normal; loose connective tissue composed of collagen fibers and adipocytes surrounded by a single row of synoviocytes was obtained in both groups. There was no synovial hyperplasia or subintimal fibrosis at any time interval in either group. The structure of the articular cartilage and cartilage cells was normal at all time intervals. Moreover, the articular cartilage was intact at all time intervals in both groups (Fig. 3) (Fig. 4).

## Discussion

In our study, we investigated the effects of dexmedetomidine on intraarticular cartilage tissue. The dexmedetomidine and saline groups were found to be similar in terms of congestion, edema, neutrophil infiltration, and chronic inflammation at all prespecified time intervals.

Intraarticular drug administration is an easily performed analgesic technique that is also very simple and easy to perform in arthroscopic knee surgery and does not require any specific or expensive medical equipment. Moreover, intraarticular drug administration yields satisfactory results, especially when used as a component of multimodal analgesia. These beneficial properties of intraarticular drug administration and the reports regarding its clinical effects in humans (prior to experimental and animal investigations), which exposed limited data about their histopathologic effects, may have inspired the performance of studies. Supporting this view, initial studies evaluating the effects of intraarticular bupivacaine did not evaluate the effects of bupivacaine on chondrocyte functions. Sun and colleagues published meta-analyses and a systematic review of 28 randomized controlled trials that assessed the analgesic efficacy of the intraarticular injection of bupivacaine after arthroscopic knee surgery [19]. They concluded that the intraarticular injection of bupivacaine relieved postoperative pain better than placebo and was statistically better than placebo in relieving postoperative pain in 24 h. Short-term adverse effects, such as sedation, nausea-vomiting and urinary retention, were reported without considering the effects of local anesthetics on chondrocyte functions that are possibly toxic, and neither of these studies reported a long-term follow-up and a long-term side-effect profile.

Recently, there have been many trials [20–23] attributing chondrolysis to intraarticular local anesthetic administration, resulting in active investigations addressing the chondrotoxicity of drugs used intraarticularly. In a retrospective cohort study, intraarticular infusion of bupivacaine was found to be associated with knee chondrolysis [23]. In another study by Breu and colleagues, it was concluded that mepivacaine, bupivacaine and ropivacaine exert toxic effects on chondrocytes in a timely manner that is dependent on the concentration and drug type [24]. In a similar controlled laboratory study, chondrocyte viability was shown to decrease significantly after administration of a single dose of 1% lidocaine in the experimental cultures compared to the control cultures [20]. In a review by Piper and colleagues, it was suggested that intraarticular local anesthetics should be used cautiously, and continuous infusions of high concentrations of local anesthetics should be avoided [25, 26].

The intraarticular use of dexmedetomidine in humans has recently gained great interest. Al-Metwalli and colleagues assessed the potential pain relief effect of intraarticular dexmedetomidine in patients undergoing arthroscopic knee procedures. They concluded that dexmedetomidine improved patient satisfaction by enhancing postoperative analgesia. In another study by Panigrahi R and colleagues, the pain relief effect of intraarticular dexmedetomidine (2 μg/kg) as an adjuvant to ropivacaine in arthroscopic surgeries proved to have superior analgesic effect in arthroscopic knee procedures and decreased the need for postoperative analgesics, without any major adverse effect, apart from the chondrocyte functions being evaluated [27]. Shaimaa FM and colleagues assessed the effects of dexmedetomidine as an adjuvant to bupivacaine on pain scores and analgesic consumption [2]. In that double-blind randomized controlled study, dexmedetomidine as an adjunct to local anesthetic was found to prolong the analgesic duration and decrease the postoperative analgesic requirement [2]. The researchers underlined the safety concerns about local adverse effects on chondrocytes and concluded that these effects should be clarified before making any recommendation about the drug [9].

A meta-analysis that included 12 RCTs involving 594 participants reported that intraarticular dexmedetomidine use decreased postoperative pain and opioid consumption in patients undergoing arthroscopic surgery [28]. The incidence of side effects was reported to be low, and this was suggested to be associated with the lack of vessels on the articular surface [28] as well as all other circumstances in which an agent was administered intraarticularly. The lack of vessels on the articular surface results in the prolonged presence of those agents in the region, leading to their prolonged activity as well as their failure to be removed. Moreover, articular cartilage does not contain tissue macrophages that would remove any apoptotic or necrotic tissues [29]. The failure to remove these tissues causes further damage, reflecting the high cellular death rates in osteoarthritic tissues [24] compared to healthy tissues after intraarticular drug administration. As acute cell death is mainly due to necrosis followed by apoptosis, and both the agent and necrotic tissues are not sufficiently removed from the site, any possible cytotoxic administration or intervention expediting the apoptotic pathway should be avoided. However, none of these studies addressing the clinical effects of the intraarticular administration of dexmedetomidine reported data on long-term effects.

To the best of our knowledge, there is only one published trial that assessed the effects of dexmedetomidine on equine chondrocytes in vitro [30]. In this study, chondrocytes isolated from healthy articular cartilage in horses [30] were treated with different concentrations (0.001–0.25 mg/ml) of dexmedetomidine for 15, 30, and 60 min [30]. Dexmedetomidine reduced cell viability in a dose-dependent manner. This effect of dexmedetomidine was evident only at concentrations of 0.175 mg/ml and 0.25 mg/ml [30]. Signs of late apoptosis and necrosis were reported [30]. That study offers recent insights into potential chondrotoxicity and warns us against the use of higher concentrations of intra-articular dexmedetomidine [30]. The concentration of dexmedetomidine, which is manufactured for human use, is 0.1 mg/ml (Precedex©), and it is nearly impossible to use higher concentrations of this drug intraarticularly in humans. We investigated the effects of this concentration (the highest concentration manufactured to be used in humans) of dexmedetomidine in our trial to determine the possible histopathological adverse reactions.

### Limitations

Sprague-Dawley rats are widely used in toxicology studies as standard models; however, articular changes in rats may not be directly comparable to those in humans and thus may not reflect the response in humans ^10^. On the other hand, the results of our study, which examined the histopathological changes in the cartilage and synovium in healthy rat knees, may not reflect any impact of dexmedetomidine on damaged knee structures or any effect of repetitive administration. In the study by Cheng et al., dexmedetomidine was investigated for its effect on osteoarthritis induced by papain in rat knees, and it was reported that dexmedetomidine improved the damage in cartilage tissue in osteoarthritis [31]. In our study, our primary outcome was not to determine the treatment effect of dexmedetomidine on cartilage tissue but to investigate the safety of this drug in healthy chondrocytes in advance. The lack of a group of rats that had chondroarthritis may be considered a limitation to our study. In the in vitro study conducted by Mancini et al., it was concluded that dexmedetomidine may cause signs of late apoptosis and necrosis at high doses, and the researchers recommended using these high doses with care for intra-articular injection [30]. In our study, we did not use such high doses, and apoptosis and necrosis were not the parameters that we evaluated for the effects of dexmedetomidine on healthy chondrocytes. These parameters may have led to more detailed information about the potential effects of dexmedetomidine on chondrocytes, which may be considered a limitation to our study.

Another major limitation of our study is the small sample size. Although the type II error was acceptable (power 80%) and we used Bonferroni correction to control type I error, a larger sample size would probably be needed to detect a smaller percentage of difference in inflammation between dexmedetomidine and saline. However, this is the first study to examine the histopathological effects of dexmedetomidine on knee joint structures, which is widely used in humans although still not approved for the intraarticular route.

Furthermore, we observed significant sedation in all groups although dexmedetomidine was injected intraarticularly, so a central analgesic effect resulting from systemic absorption cannot be excluded [3]; however, the plasma concentration of the analgesics used was not measured.

## Conclusion

According to the short-term results of our in vivo, experimental animal study, the intraarticular use of dexmedetomidine seems safe in relation to histopathological analyses. However, it is crucial to consider the ethical, legal and financial aspects of the complications of intra-articular drug administration of an agent that is not approved for the intraarticular route.

## Data Availability

The datasets used and/or analysed during the current study available from the corresponding author on reasonable request.
